# The causes of preterm neonatal deaths in India and Pakistan (PURPOSe): a prospective cohort study

**DOI:** 10.1016/S2214-109X(22)00384-9

**Published:** 2022-10-11

**Authors:** Sangappa M Dhaded, Sarah Saleem, Shivaprasad S Goudar, Shiyam Sunder Tikmani, Kay Hwang, Gowdar Guruprasad, Gayathri H Aradhya, Varun B Kusagur, Lingaraja Gowda C Patil, S Yogeshkumar, Manjunath S Somannavar, Sayyeda Reza, Sana Roujani, Jamal Raza, Haleema Yasmin, Anna Aceituno, Lindsay Parlberg, Jean Kim, Janet Moore, Carla M Bann, Robert M Silver, Robert L Goldenberg, Elizabeth M McClure, PURPOSe study group, Shivaprasad Goudar, Sangappa M Dhaded, Mahantesh B Nagmoti,, Manjunath S Somannavar, S Yogeshkumar, Sheetal Harakuni, Gowdar Guruprasad, Gayathri H Aradhya, Naveen Nadig, Varun Kusgur, Chaitali R Raghoji, B Sarvamangala, Veena Prakash,, Upendra Kumar Joish, G K Mangala, K S Rajashekhar, K Byranahalli Sunilkumar, Vardendra Kulkarni, ES Siddartha, Lingaraja Gowda C Patil, Sneharoopa Pujar, Shobha Dhananjaya, TS Nagaraj, MU Jeevika, Reddy R Harikiran, Sarah Saleem, Shiyam Sunder Tikmani, Afia Zafar, Imran Ahmed, Zeeshan Uddin, Najia Ghanchi, Sana Roujani, Shabina Ariff, Lumaan Sheikh, Waseem Mirza, Haleema Yasmin, Jamal Raza, Jai Prakash, Furqan Haider, Anna Aceituno, Lindsay Parlberg, Janet L Moore, Kay Hwang, Suchita Parepelli, Jean Kim, Carla Bann, Elizabeth McClure, Robert Goldenberg, Robert Silver

**Affiliations:** a KLE Academy of Higher Education and Research, JN Medical College, Belagavi, Karnataka, India; b Department of Community Health Sciences, Aga Khan University, Karachi, Pakistan; c Research Triangle Institute International, Durham, NC, USA; d Bapuji Educational Association's JJM Medical College, Davangere, Karnataka, India; e National Institute of Child Health, Karachi, Pakistan; f Jinnah Postgraduate Medical Centre, Karachi, Pakistan; g Department of Obstetrics and Gynecology, University of Utah School of Medicine, Salt Lake City, UT, USA; h Department of Obstetrics and Gynecology, Columbia University, New York, NY, USA

## Abstract

**Background:**

Preterm birth remains the major cause of neonatal death worldwide. South Asia contributes disproportionately to deaths among preterm births worldwide, yet few population-based studies have assessed the underlying causes of deaths. Novel evaluations, including histological and bacteriological assessments of placental and fetal tissues, facilitate more precise determination of the underlying causes of preterm deaths. We sought to assess underlying and contributing causes of preterm neonatal deaths in India and Pakistan.

**Methods:**

The project to understand and research preterm pregnancy outcomes and stillbirths in South Asia (PURPOSe) was a prospective cohort study done in three hospitals in Davangere, India, and two hospitals in Karachi, Pakistan. All pregnant females older than 14 years were screened at the time of presentation for delivery, and those with an expected or known preterm birth, defined as less than 37 weeks of gestation, were enrolled. Liveborn neonates with a weight of 1000 g or more who died by 28 days after birth were included in analyses. Placentas were collected and histologically evaluated. In addition, among all neonatal deaths, with consent, minimally invasive tissue sampling was performed for histological analyses. PCR testing was performed to assess microbial pathogens in the placental, blood, and fetal tissues collected. An independent panel reviewed available data, including clinical description of the case and all clinical maternal, fetal, and placental findings, and results of PCR bacteriological investigation and minimally invasive tissue sampling histology, from all eligible preterm neonates to determine the primary and contributing maternal, placental, and neonatal causes of death.

**Findings:**

Between July 1, 2018, and March 26, 2020, of the 3470 preterm neonates enrolled, 804 (23%) died by 28 days after birth, and, of those, 615 were eligible and had their cases reviewed by the panel. Primary maternal causes of neonatal death were hypertensive disease (204 [33%] of 615 cases), followed by maternal complication of pregnancy (76 [12%]) and preterm labour (76 [11%]), whereas the primary placental causes were maternal and fetal vascular malperfusion (172 [28%] of 615) and chorioamnionitis, funisitis, or both (149 [26%]). The primary neonatal cause of death was intrauterine hypoxia (212 [34%] of 615) followed by congenital infections (126 [20%]), neonatal infections (122 [20%]), and respiratory distress syndrome (126 [20%]).

**Interpretation:**

In south Asia, intrauterine hypoxia and congenital infections were the major causes of neonatal death among preterm babies. Maternal hypertensive disorders and placental disorders, especially maternal and fetal vascular malperfusion and placental abruption, substantially contributed to these deaths.

**Funding:**

Bill & Melinda Gates Foundation.

## Introduction

Most neonates who are born preterm in high-income countries now survive; however, preterm birth remains the major contributor to neonatal death globally.^1^, ^2^, ^3^ The Asian region contributes to more than half of all preterm births, and India alone contributes to nearly a quarter.^3^ Although advances have been made in reducing child mortality in this region, the number of deaths attributed to preterm birth remains disproportionately high.^4^ So far, complications of prematurity have been classified as the major cause of child deaths, with little differentiation among the sub-causes of death in preterm babies, especially in low-income and middle-income countries (LMICs). Ambiguity persists because the cause-of-death estimates have frequently been based on verbal autopsy or clinical evaluation.^5^, ^6^, ^7^ These methods lack specificity, especially for differentiating intrapartum hypoxia from other respiratory disorders and from infectious conditions, which are among the most common causes of neonatal deaths.

To help improve accuracy of cause-of-death estimation, minimally invasive tissue sampling (MITS) can facilitate histological and microbiological evaluations of selected tissues.^8^, ^9^, ^10^ For preterm neonatal deaths, histological evaluation of the placenta might also have an important role in determining the cause of death because various placental conditions can contribute to preterm neonatal deaths.^11^ However, placentas are rarely evaluated as part of neonatal death investigations.

**Research in context**

**Evidence before this study**

We searched PubMed for research articles published in English between May 2, 2012, and May 1, 2022, using the terms ”neonatal death” OR “fetal death” OR “perinatal mortality” OR “preterm birth” AND “low- and middle-income countries” OR “developing countries” OR “Asia”. In low-income and middle-income countries (LMICs), preterm birth remains a major contributor to neonatal mortality. Although childhood mortality has declined, neonatal mortality has not shown a similar decrease. The ability to address neonatal mortality in preterm infants is hampered by the absence of accurate cause-of-death data in LMICs. When causes are available, a substantial proportion of the reports from LMICs have been based on verbal autopsy, with the deaths attributed to prematurity without differentiating the sub-causes. Recent advances in cause-of death investigations, including minimally invasive tissue sampling for histological examination and pathogen identification by PCR, have improved the ability to determine more granular understanding of the causes of death, especially in LMICs. However, so far, few studies have examined the underlying causes of death among preterm births in LMICs.

**Added value of this study**

The project to understand and research preterm pregnancy outcomes and stillbirths in South Asia (PURPOSe), one of the largest prospective studies of preterm neonatal mortality from south Asia so far, included detailed assessment of all preterm neonatal deaths, including placental assessment, physical examination, and histological evaluation of neonatal tissues. Placental and fetal tissues were also tested for a wide range of pathogens by PCR. Expert panelists reviewed all available data presented in standardised format to determine the primary and contributing maternal, placental, and fetal causes based on the tenth revision of the WHO International Classification of Diseases Perinatal Mortality classification.

**Implications of all the available evidence**

The techniques used for this study, which was conducted in hospitals with a range of resources, can inform future efforts to more accurately determine cause of death. Our results suggest that to achieve substantial reductions in preterm neonatal deaths, more attention should be paid to improving obstetric care and among preterm births in LMIC settings. The major finding from this study is that intrauterine hypoxia is among the most important causes of preterm neonatal mortality. Improving obstetric care around delivery as well as neonatal resuscitation should have an important effect on neonatal deaths resulting from intrauterine hypoxia.

Two recent studies used MITS to evaluate causes of death in neonates. One study from Ethiopia, which was done in preterm newborn babies admitted to a neonatal intensive care unit, found the major causes of death to be respiratory distress syndrome (45%), neonatal infections (30%), and asphyxia (14%).^12^ The Child Health and Mortality Surveillance (CHAMPS) study, which was done in seven LMICs (primarily in African sites), found the leading cause of neonatal death to be complications of prematurity (42%).^13^ Both studies did not routinely evaluate placental histology.

The main aim of the project to understand and research preterm pregnancy outcomes and stillbirths in South Asia (PURPOSe) was to improve the accuracy in determining the specific causes of death in preterm neonates born in India and Pakistan.^14^ Although PURPOSe included causes of death for both preterm liveborn deaths and stillbirths, we report the results of the cause-of-death analyses for preterm neonates.

## Methods

### Study design and population

PURPOSe was a prospective cohort study done in two south Asian sites, one in Davangere, India (three hospitals providing public and private services), and one in Karachi, Pakistan (one public maternity and one children's referral hospital). Detailed study methodology has been published.^14^, ^15^, ^16^

At participating study hospitals, research staff screened all pregnant females older than 14 years at the time of presentation for delivery. Pregnant females with an expected or known preterm birth, defined as less than 37 weeks of gestation, were approached for enrolment at the time of admission to hospital. Girls aged 14 years or younger and those who did not provide consent were excluded. Preterm neonates with a weight of 1000 g or more who died by 28 days after birth were included in analyses.

The study was reviewed and approved by the ethics review committees at participating sites: the Aga Khan University (Karachi, Pakistan), KLE Academy of Higher Education and Research (Belagavi, India), JJM Medical College (Davangere, India), and Research Triangle Insititute International (Durham, NC, USA). All women provided informed written consent for themselves and their neonate (or neonates) before participation in the study.

### Data collection

At the time of delivery, study staff recorded results of the physical examination, medical and obstetric history, and the clinical status and procedures conducted during the current delivery. Gestational age was based on clinical ultrasound or, when unavailable, was determined by last menstrual period and by clinical assessment based on the American College of Obstetricians and Gynecologists recommendations for estimating pregnancy due date and gestational age.^17^ The placenta and umbilical cord blood were collected for all deliveries.^18^ Placentas were collected and evaluated in a fresh state. Tissue samples were taken for microscopic examination from the umbilical cord, fetal membranes, and the placental disc as well as additional samples of any lesions present.^19^ Trained pathologists did a gross evaluation and evaluation of the histopathology of placentas using the Amsterdam Consensus criteria.^20^

Newborn babies were weighed and their lengths were measured after delivery by trained staff. In addition, for neonatal deaths, a physical examination was conducted at the time of death. With additional consent, trained study staff conducted a MITS, which included needle biopsy using asceptic techniques to collect at least two tissues samples in each of lung, liver, and brain tissues, as well as 8 mL of blood and cerebrospinal fluid, which were collected using standardised procedures.^21^ MITS samples were assessed using histological and molecular microbiological methods.^22^ The TaqMan Array Card (ThermoFisher, Waltham, MA, USA) PCR testing, which was developed and validated by the US Centers for Disease Control and Prevention for 75 pathogens, was used to evaluate organisms in the fetal tissues, umbilical cord, membrane, and placental tissues.^23^ At the Indian site, with additional consent, a standardised complete autopsy was also performed. For infants who died after discharge, a verbal autopsy was performed in sites in India and Pakistan.

### Cause-of-death determination

The cause (or causes) of death for preterm infants with a birthweight of 1000 g or more was determined by an independent panel in India and a separate one in Pakistan. The panelists determined the primary maternal, placental, and neonatal causes of death and the contributing causes using standard procedures described in detail elsewhere.^15^, ^16^ These panels comprised obstetricians, paediatricians, pathologists, microbiologists, and other clinicians.

For each case, a pair of panelists, who were not directly involved with the conduct of the study, reviewed the cases. A brief clinical description of the case and all positive clinical maternal, fetal, and placental findings, and results of the PCR bacteriological investigation and MITS histology were included in each report. Additionally, reference weight measures including the 10th percentile for gestational age based on the INTERGROWTH-21st criteria^24^ and mean placental weight for gestational age were presented in a standardised data report. Each pair of panelists reviewed their cases, with any discrepancies resolved through discussion among the team. Causes of death used nomenclature from the tenth revision of the International Classification of Diseases Perinatal Mortality system.^25^ Using this system, infectious causes of death were identified as congenital infections (ie, those acquired before delivery) and neonatal infections (ie, those determined to have been acquired following delivery).

### Statistical analysis

The planned study sample size was 350 deceased preterm neonates in both India and Pakistan to provide sufficient precision to detect causes of death that contributed to 20% or more of the deaths with 95% power. To achieve the required sample size, based on reported mortality rates**,** we attempted to screen and enrol all pregnant females admitted to study hospitals in preterm labour or scheduled for a preterm delivery. All data were entered and reviewed at each site. Descriptive analyses were performed using SAS (version 9.4).

### Role of the funding source

The funder of the study had no role in study design, data collection, data analysis, data interpretation, or writing of the report.

## Results

Between July 1, 2018, and March 26, 2020, 4030 liveborn neonates of women presenting in preterm labour or for preterm delivery (ie, born before 37 weeks of gestation) were screened and 3636 of those neonates were eligible; consent was obtained from the mothers of 3470, and they were enrolled in the study (figure). Of the preterm neonates enrolled, 615 died by the age of 28 days and had a birthweight of 1000 g or more, and their deaths were reviewed by the independent panel. Among the deaths reviewed, 615 had clinical and physical evaluations completed, 582 had a placental evaluation (histology and PCR), 246 had MITS (histology and PCR), 105 had a verbal autopsy, and 64 had a full perinatal autopsy. Because of multiple births, these neonates came from 559 pregnancies, with a cause-of-death review included in the analyses of cause of preterm neonatal death.

**Figure:**
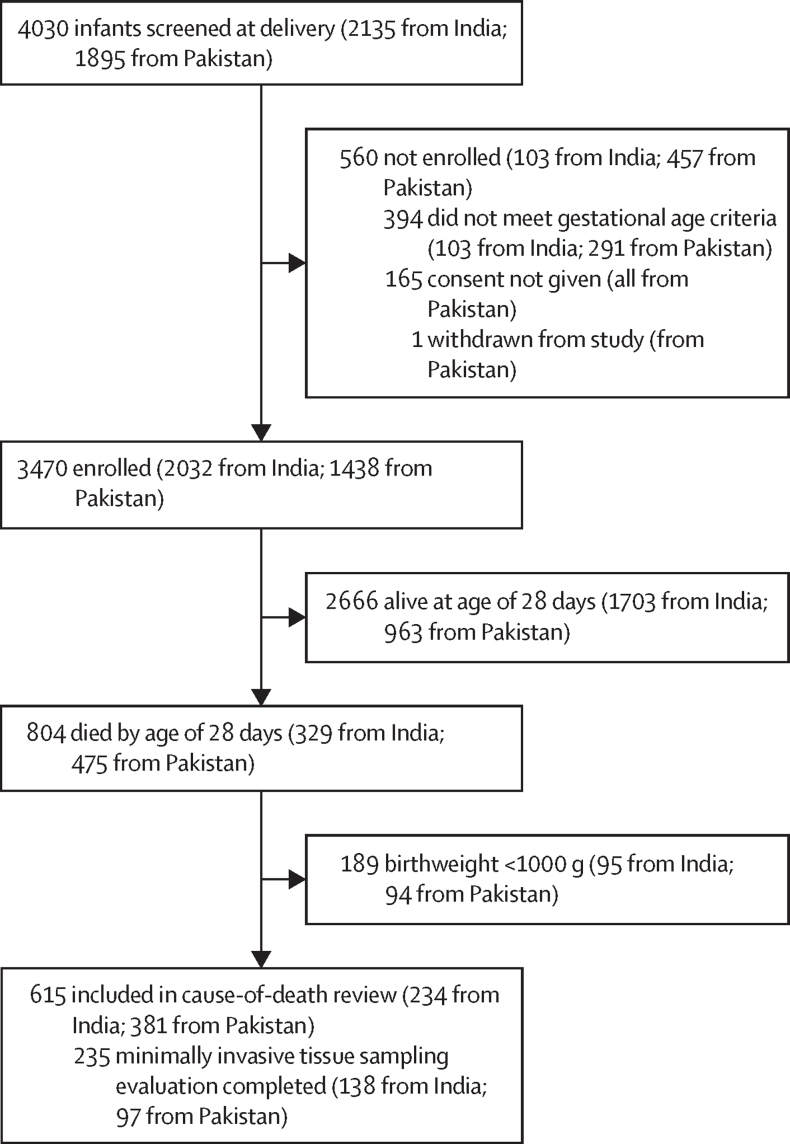
Study profile

68 (11%) of the 615 reviewed cases were 20 to <28 weeks gestational age, 248 (40%) were 28 to <32 weeks gestational age, and 299 (49%) were 32 to <37 weeks (table 1). Furthermore, 288 (47%) neonates weighed 1000–1499 g, 289 (47%) weighed 1500 to <2500 g, and 38 (6%) weighed 2500 g or more. 132 (22%) preterm neonates were diagnosed with fetal growth restriction. 123 (20%) of the deaths occurred within 24 h of delivery, 247 (40%) at 1–3 days, 105 (17%) at 4–6 days, and 136 (22%) at 7–28 days. Among the neonatal deaths, 484 (79%) of the infants had been admitted to the neonatal intensive care unit: 197 (84%) of 234 in India and 287 (75%) of 381 in Pakistan.

**Table 1:** Neonatal death characteristics

		**Overall (n=615)**	**India (n=234)**	**Pakistan (n=381)**
Gestational age, weeks				
	20 to <28	68 (11%)	20 (9%)	48 (13%)
	28 to <32	248 (40%)	80 (34%)	168 (44%)
	32 to <37	299 (49%)	134 (57%)	165 (43%)
Birthweight, g				
	1000–1499	288 (47%)	135 (58%)	153 (40%)
	1500–2499	289 (47%)	88 (38%)	201 (53%)
	≥2500	38 (6%)	11 (5%)	27 (7%)
Fetal growth restriction (IUGR)*		132 (22%)	79 (34%)	53 (14%)
Admitted to NICU		484 (79%)	197 (84%)	287 (75%)
Age at death, days				
	<1	123 (20%)	54 (23%)	69 (18%)
	1–3	247 (40%)	78 (33%)	169 (44%)
	4–6	105 (17%)	43 (18%)	62 (16%)
	≥7	136 (22%)	59 (25%)	77 (20%)
	Missing	4 (1%)	0	4 (1%)

Data are n (%). Percentages might not sum to 100% as a result of rounding. IUGR=intrauterine growth retardation. NICU=neonatal intensive care unit.

* Fetal growth restriction is defined as a birthweight of less than the INTERGROWTH-21st^24^ 10th percentile weight, which is not available for gestational age of less than 24 weeks or 43 weeks or more, and for fetuses missing sex or birthweight data.

248 (44%) of the 559 women with neonates in the study were aged 20–25 years at the time of delivery, and 191 (34%) were 26–30 years (table 2). 184 (33%) of 556 women had no formal schooling, which was more common in the Pakistani sites than in the Indian sites, whereas 344 (62%) of 556 mothers had 1–12 years of schooling. 19 (3%) women were employed outside the home, and the proportions were similar in both sites. 186 (33%) women were primagravidas, 287 (52%) had one to three previous pregnancies, and 86 (15%) had more than three previous pregnancies.

**Table 2:** Maternal baseline characteristics

		**Overall (n=559)**	**India (n=214)**	**Pakistan (n=345)**
Multiple pregnancy		43 (8%)	13 (6%)	30 (9%)
Maternal age at delivery, years				
	>14 to 20	38 (7%)	17 (8%)	21 (6%)
	>20 to 25	248 (44%)	116 (54%)	132 (38%)
	>25 to 30	191 (34%)	63 (29%)	128 (37%)
	>30	82 (15%)	18 (8%)	64 (19%)
Maternal education				
	No formal schooling	184/556 (33%)	25/211 (12%)	159 (46%)
	1–8 years	158/556 (28%)	72/211 (34%)	86 (25%)
	9–12 years	186/556 (33%)	95/211 (45%)	91 (26%)
	>12 years	28/556 (5%)	19/211 (9%)	9 (3%)
Maternal occupation				
	Homemaker	540 (97%)	205 (96%)	335 (97%)
	Government or company	2 (<1%)	1 (<1%)	1 (<1%)
	Self-employed	1 (<1%)	1 (<1%)	0
	Farmer	6 (1%)	5 (2%)	1 (<1%)
	Other	10 (2%)	2 (1%)	8 (2%)
Gravida				
	0	186 (33%)	82 (38%)	104 (30%)
	1–3	287 (51%)	123 (57%)	164 (48%)
	>3	86 (15%)	9 (4%)	77 (22%)

Data are n (%) or n/N (%). Percentages might not sum to 100% as a result of rounding.

Hypertensive disorders of pregnancy were the primary maternal cause of death in 204 (33%) of the 615 cases, followed by maternal complications of pregnancy (76 [12%]) and preterm labour (70 [11%]; table 3). No primary maternal cause of death was identified for 159 (26%) cases. Important maternal conditions that contributed to the neonatal deaths included hypertensive disorders in pregnancy (25 [4%] cases), maternal medical and surgical conditions (130 [21%]; eg, hyperthyroidism or appendicitis), maternal complications of pregnancy (106 [17%]), and preterm labour (64 [10%]). No contributory maternal cause was found in 292 (47%) cases.

**Table 3:** Primary and contributing maternal conditions related to neonatal death

	**Primary maternal cause**			Contributing maternal causes*		
	Overall (n=615)	India (n=234)	Pakistan (n=381)	Overall (n=615)	India (n=234)	Pakistan (n=381)
Eclampsia, pre-eclampsia, or other hypertensive disorder	204 (33%)	77 (33%)	127 (33%)	25 (4%)	13 (6%)	12 (3%)
Other maternal complications of pregnancy	76 (12%)	30 (13%)	46 (12%)	106 (17%)	43 (18%)	63 (17%)
Preterm labour	70 (11%)	33 (14%)	37 (10%)	64 (10%)	31 (13%)	33 (9%)
Other complications of labour and delivery	31 (5%)	11 (5%)	20 (5%)	22 (4%)	12 (5%)	10 (3%)
Maternal infectious and parasitic disease	30 (5%)	4 (2%)	26 (7%)	36 (6%)	4 (2%)	32 (8%)
Diabetes	9 (1%)	2 (1%)	7 (2%)	16 (3%)	6 (3%)	10 (3%)
Severe maternal infection	7 (1%)	2 (1%)	5 (1%)	4 (1%)	0	4 (1%)
Other maternal medical and surgical conditions	29 (5%)	3 (1%)	26 (7%)	130 (21%)	14 (6%)	116 (30%)
No maternal cause identified	159 (26%)	72 (31%)	87 (23%)	292 (47%)	132 (56%)	160 (42%)

Data are n (%).

* More than one contributing cause possible.

The placenta was collected and evaluated for 582 (95%) of the 615 cases (table 4). Primary placental causes of death determined by the panel were maternal or fetal vascular malperfusion (172 [28%] of 615 cases); chorioamnionitis, funisitis, or both (149 [25%]); and placental abruption (38 [6·5%]). No primary placental cause of death was found in 176 (30%) cases. Common contributory placental causes of death included chorioamnionitis, funisitis, or both (83 [13·5%] cases) and maternal and fetal vascular malperfusion (70 [11%]). No contributing placental causes of death were found in 422 (69%) cases, with similar distributions found in the Indian and Pakistani sites.

**Table 4:** Primary and contributing placental conditions related to neonatal death

	**Primary placental cause**			Contributing placental causes*		
	Overall (n=615)	India (n=234)	Pakistan (n=381)	Overall (n=615)	India (n=234)	Pakistan (n=381)
Placentas evaluated	582 (95%)	230 (98%)	352 (92%)	582 (95%)	230 (98%)	352 (92%)
Maternal or fetal vascular malperfusion	172/582 (30%)	80/230 (35%)	92/352 (26%)	70/582 (12%)	40/230 (17%)	30/352 (9%)
Chorioamnionitis, funisitis, or both	149/582 (26%)	44/230 (19%)	105/352 (30%)	83/582 (14%)	24/230 (10%)	59/352 (17%)
Placental abruption	38/582 (7%)	15/230 (7%)	23/352 (7%)	9/582 (2%)	5/230 (2%)	4/352 (1%)
Placenta previa	24/582 (4%)	3/230 (1%)	21/352 (6%)	3/582 (1%)	1/230 (<1%)	2/352 (1%)
Prolonged membrane rupture	1/582 (<1%)	1/230 (<1%)	0	6/582 (1%)	5/230 (2%)	1/352 (<1%)
Umbilical cord complication	0	0	0	3/582 (1%)	0	3/352 (1%)
Other complications of placenta, umbilical cord, and membranes	28/582 (5%)	6/230 (3%)	22/352 (6%)	36/582 (6%)	16/230 (7%)	20/352 (6%)
No placental cause identified	176/582 (30%)	84/230 (37%)	92/352 (26%)	422/582 (73%)	152/230 (66%)	270/352 (77%)

Data are n (%) or n/N (%).

* More than one contributing cause possible.

314 (51%) of the 615 deaths were attributed to non-infectious conditions (table 5). Among the non-infectious causes of death, the leading cause was intrauterine hypoxia (212 [68%] of 314 cases), followed by respiratory distress syndrome (63 [20%]) and congenital malformations, deformations, and chromosomal abnormalities (28 [9%]). Congenital infections (126 [20%] of 615) and neonatal infections (122 [20%]) were identified as the primary cause of death for a substantial proportion of the cases. The most common organisms found in an internal organ by PCR for these deaths included *Acinetobacter baumanii*, *Klebsiella pneumoniae, Escherichia coli*, and shigella species (data not shown). Group B streptococcus was rarely found (four [2%] of 246 cases) by PCR analyses in any internal organ. For 53 (9%) of 615 cases, no primary neonatal cause of death could be identified.

**Table 5:** Primary and non-primary neonatal cause of neonatal death

	**Primary neonatal cause**			Contributing neonatal causes*		
	Overall (n=615)	India (n=234)	Pakistan (n=381)	Overall (n=615)	India (n=234)	Pakistan (n=381)
Non-infectious conditions	314 (51%)	113 (48%)	201 (53%)	387 (63%)	174 (74%)	213 (56%)
Intrauterine hypoxia	212/314 (68%)	57/113 (50%)	155/201 (77%)	138/387 (36%)	67/174 (39%)	71/213 (33%)
Respiratory distress syndrome (hyaline membrane disease)	63/314 (20%)	30/113 (27%)	33/201 (16%)	170/387 (44%)	108/174 (62%)	62/213 (29%)
Congenital malformations, deformations, and chromosomal abnormalities	28/314 (9%)	19/113 (17%)	9/201 (4%)	29/387 (7%)	14/174 (8%)	15/213 (7%)
Pulmonary haemorrhage	6/314 (2%)	5/113 (4%)	1/201 (<1%)	21/387 (5%)	19/174 (11%)	2/213 (1%)
Necrotising entercolitis	2/314 (1%)	2/113 (2%)	0	8/387 (2%)	7/174 (4%)	1/213 (<1%)
Intraventricular haemorrhage of the neonate	1/314 (<1%)	0	1/201 (<1%)	24/387 (6%)	20/174 (11%)	4/213 (2%)
Intracranial haemorrhage of the neonate unspecified	1/314 (<1%)	0	1/201 (<1%)	5/387 (1%)	5/174 (3%)	0
Meconium aspiration	1/314 (<1%)	0	1/201 (<1%)	3/387 (1%)	0	3/213 (1%)
Hypothermia (neonatal cold injury)	0	0	0	108/387 (28%)	3/174 (2%)	105/213 (49%)
Apnoea of prematurity	0	0	0	9/387 (2%)	9/174 (5%)	0
Disseminated intravascular coagulation	0	0	0	8/387 (2%)	8/174 (5%)	0
Neonatal anaemia	0	0	0	6/387 (2%)	4/174 (2%)	2/213 (1%)
Birth trauma	0	0	0	3/387 (1%)	1/174 (1%)	2/213 (1%)
Kernicterus	0	0	0	1/387 (<1%)	0	1/213 (<1%)
Congestive heart failure	0	0	0	1/387 (<1%)	0	1/213 (<1%)

Data are n (%) or n/N (%).

* More than one contributing cause possible.

To explore the inter-relationship between the most common cause of death from each category, we evaluated the primary cause of death from each domain (appendix). Among preterm neonates who died from the most common neonatal cause, intrauterine hypoxia, 91 (43%) of 212 also had maternal hypertensive disease and 68 (32%) also had placental maternal malperfusion as causes of death.

## Discussion

The PURPOSe study represents one of the largest prospective studies so far on the causes of preterm neonatal death from south Asia. Hypertensive disease was the primary maternal cause of nearly a third of the deaths. Placental and umbilical cord conditions were an important primary or contributing factor, mostly involving maternal and fetal placental malperfusion and chorioamnionitis, funisitis, or both. Among neonatal causes of death, about half were non-infectious, whereas about 40% were infectious.

The most common primary neonatal cause of death determined by the panel was intrauterine hypoxia, followed by congenital and neonatal infections, and finally respiratory distress syndrome. We observed that respiratory distress syndrome was designated as the primary cause of death less frequently than is generally reported. We believe that preterm infants with respiratory distress are often diagnosed with respiratory distress syndrome, but often without a confirmatory chest x-ray or presence of hyaline membranes on lung histology. If those findings are absent, other reasons for respiratory distress, including birth asphyxia and sepsis, are likely and can often be elucidated with more information, as was seen in the PURPOSe study. Because intrauterine hypoxia is the most common final pathway leading to preterm neonatal death, prevention and treatment of this condition should be a major focus of efforts to reduce preterm neonatal mortality.^26^ Improvements in both obstetric care and neonatal resuscitation will probably be required to reduce mortality from this condition.^27^

This study had prospective data collection in two countries with a range of quality of care, and with high-quality historical, histological, and microbial evaluation to inform the assessments. The inclusion of placental pathology is often crucial in determining cause of preterm neonatal deaths but is rarely obtained. Independent panelists had available a case report with key investigations summarised. Finally, the primary findings across the sites were similar. Another strength was the inclusion of maternal, placental, and neonatal causes of death to highlight the inter-relationship of conditions. For example, if a mother had hypertension, the placenta had vascular malperfusion, and the neonate had birth asphyxia, specific inclusion of each of these areas facilitated an understanding of the contribution of the maternal, placental, and neonatal causes of this death.

One of the study constraints was that maternal conditions were only collected at enrolment, usually before delivery, so there were potential limitations regarding the data on maternal conditions and gestational age ascertainment. Another limitation was the quality of data on conditions among those infants discharged home before death; however, this represented a small proportion of all neonatal deaths evaluated. Although the causes of preterm neonatal deaths were generally similar in India and Pakistan, we are aware that these findings might not be representative of other sites in India and Pakistan, in sites in other Asian countries, or in other LMICs worldwide. For this reason, it is important to determine causes of preterm neonatal death in multiple locations so the appropriate interventions can be introduced.

In contrast to high-resource settings, where almost all preterm newborn babies survive, prematurity remains a leading cause of neonatal death globally. Furthermore, in low-resource settings, complications of prematurity are often listed as the cause of death, with little understanding of the factors that could inform programmes to reduce these deaths. In PURPOSe, infants who were late preterm, and would be viable in high-income countries, comprised the majority of the preterm neonatal deaths. We found that maternal hypertensive disorders together with intrauterine hypoxia were the main maternal contributors to these deaths. Congenital and newborn infections were also important causes of death. Continuing to improve our understanding of causes of preterm neonatal deaths in these settings is important to developing effective strategies to reduce this important contributor to global infant mortality.

## Data sharing

The study protocol and consent and case report forms are available from the corresponding author upon request. With investigator support, after approval of a proposal and a signed data use agreement, de-identified data from this paper will be available upon reasonable request from the corresponding author.

## Declaration of interests

We declare no competing interests.
