# Supplementary material for: The causes of preterm neonatal deaths in India and Pakistan (PURPOSe): a prospective cohort study
Source: Lancet Glob Health. 2022 Oct 11;10(11):e1575–81. doi: 10.1016/S2214-109X(22)00384-9 (PMC9579353; doi:10.1016/S2214-109X(22)00384-9)
Supplement: Supplementary appendix [file mmc1.pdf]

THE LANCET
Global Health

Supplementary appendix

This appendix formed part of the original submission and has been peer reviewed. We post it as supplied by the authors.

Supplement to: Dhaded SM, Saleem S, Goudar SS, et al. The causes of preterm neonatal deaths in India and Pakistan (PURPOSe): a prospective cohort study. *Lancet Glob Health* 2022; **10:** e1575–81.

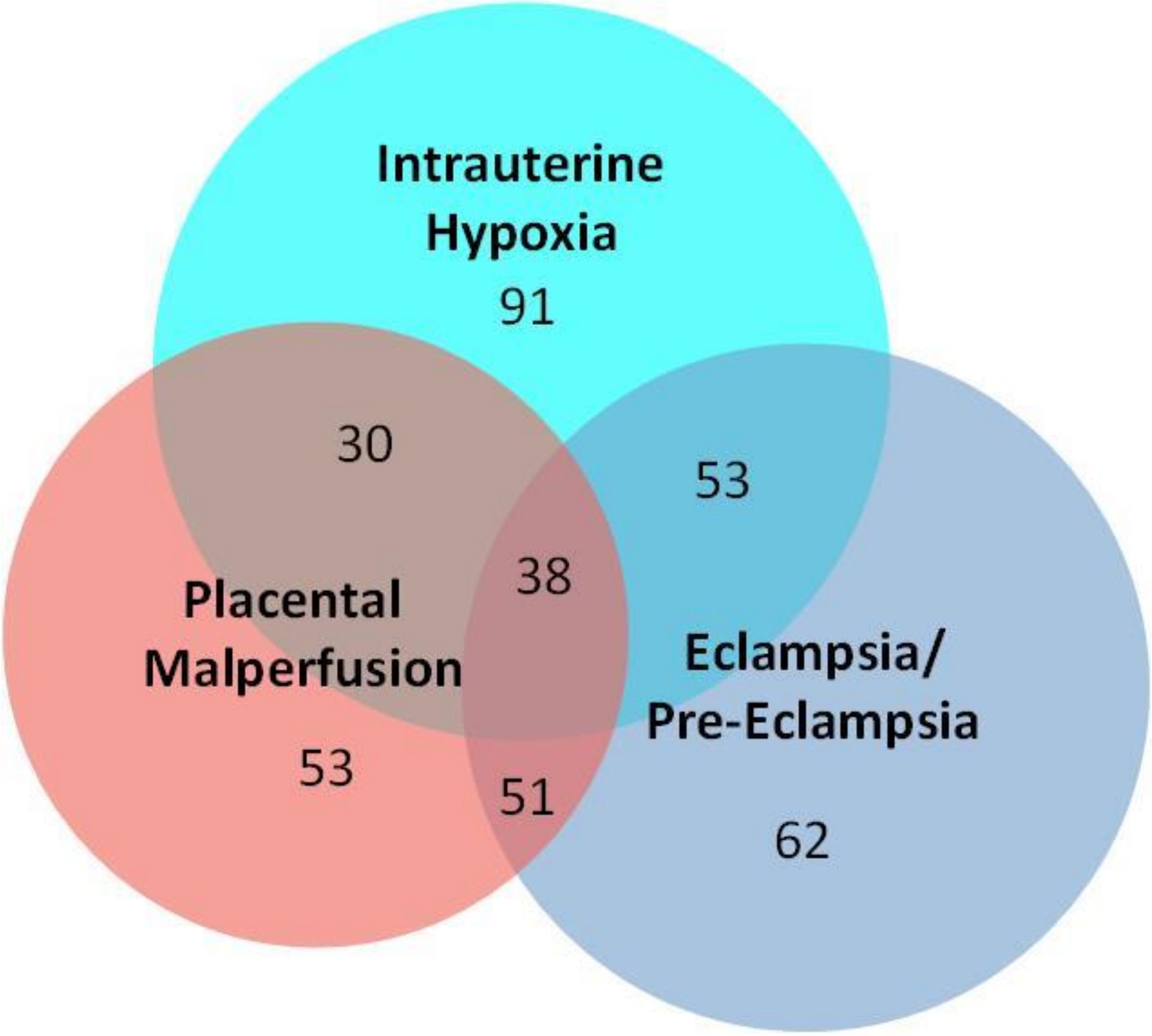

Appendix. Overlap of primary neonatal, placental and maternal cause of death in preterm neonatal deaths in PURPOSe
